# Nanosystems for oxidative stress regulation in the anti-inflammatory therapy of acute kidney injury

**DOI:** 10.3389/fbioe.2023.1120148

**Published:** 2023-02-09

**Authors:** Yue Wang, Hong Jiang, Longyao Zhang, Peng Yao, Shaoqing Wang, Qian Yang

**Affiliations:** ^1^ The Second Affiliated Hospital of Chengdu Medical College, China National Nuclear Corporation 416 Hospital, Chengdu, Sichuan, China; ^2^ Center of Scientific Research, Chengdu Medical College, Chengdu, Sichuan, China

**Keywords:** acute kideny injury, anti-inflammtory, oxidative stress, ROS scavenging, nanosystem

## Abstract

Acute kidney injury (AKI) is a clinical syndrome that results from a rapid decline in renal structure or renal functional impairment with the main pathological feature of sublethal and lethal damage to renal tubular cells. However, many potential therapeutic agents cannot achieve the desired therapeutic effect because of their poor pharmacokinetics and short retention time in the kidneys. With the recent emergence and progress of nanotechnology, nanodrugs with unique physicochemical properties could prolong circulation time, enhance efficient targeted delivery, and elevate the accumulation of therapeutics that can cross the glomerular filtration barrier and indicate comprehensive application prospects in the prevention and treatment of AKI. In this review, various types of nanosystems (such as liposomes, polymeric nanosystems, inorganic nanoparticles and cell-derived extracellular vesicles) are designed and applied to improve the pharmacokinetics of drug formation, which could further relieve the burden on the kidneys caused by the final cumulative dose of drugs in conventional treatments. Moreover, the passive or active targeting effect of nanosystems can also reduce the total therapeutic dose and off-target adverse effects on other organs. Nanodelivery systems for treating AKI that alleviate oxidative stress-induced renal cell damage and regulate the inflammatory kidney microenvironment are summarized.

## 1 Introduction

Acute kidney injury (AKI) is a clinical syndrome that results from a rapid decline in renal structure or renal functional impairment. It is considered a new global health problem due to its high incidence and rapid progression as well as its high mortality and disability rates (Kellum et al., 2021). Clinical causes include renal ischemia‒reperfusion (IR), nephrotoxic substances, sepsis, glomerulonephritis or interstitial nephritis, and extrarenal (such as prostatic hypertrophy) or intrarenal (such as kidney stones and thrombosis). The main pathological feature of AKI is sublethal and lethal damage to renal tubular cells. After AKI, the extant tubular cells undergo dedifferentiation, proliferation, and migration followed by redifferentiation to repair damaged tubular cells, which can be completely restored to intact tubules after mild injury (Sancho-Martínez et al., 2015). Moreover, severe or recurrent AKI often leads to abnormal repair of the kidney and renal fibrosis, accelerates renal function decline, and ultimately results in chronic kidney disease (CKD) and end-stage renal disease (ESRD) (Kaballo et al., 2017). Presently, the clinical treatment for AKI is mainly focused on supportive and symptomatic management as well as kidney transplantation. However, efficacy is still limited. Therefore, the development of a targeted therapeutic approach based on the pathophysiological mechanism of AKI has become a research Frontier and a hot topic (Gonsalez et al., 2019; Guo et al., 2019).

Nephron is composed of renal corpuscles and tubules. It is the basic structural and functional unit of the kidney to form urine. The renal corpuscles are composed of spherical glomeruli and renal sacs wrapped outside the glomeruli. The renal tubules are outside the renal sacs. The glomerulus is divided into three layers. The inner layer is the endothelial cell layer, which is a flat cell attached to the basement membrane of the glomerulus. There are numerous pores with a diameter of about 70–100 nm on it (Akilesh et al., 2008). The pores have a thin membrane. Such a structure allows larger molecules to easily pass through; The middle layer is the glomerular basement membrane, which is the main part of controlling the size of filtration molecules and the main part of mechanical barrier; The outer layer is the epithelial cell layer, namely the podocyte, which is the terminal differentiated cell. A variety of slit membrane proteins are intercalated with each other, forming a molecular sieve barrier that can prevent the leakage of medium and large molecular weight proteins. There is mesangial tissue between glomerular capillaries, including mesangial cells and mesangial matrix. Particles with a diameter of up to 5–7 nm under normal regulation can be filtered through the glomerulus and reach the top of the proximal tubular cells. The renal tubules are composed of proximal tubules, thin segments, distal tubules and connecting tubules. The proximal tubules are responsible for the active transport of endogenous and exogenous substances in the renal cells. The uptake of small molecules such as glucose, peptides, neurotransmitters and some large protein molecules all use active transport. Using this property to deliver drugs to proximal tubular cells through receptor-mediated endocytosis is a common delivery method of nanodrugs.

**FIGURE 1:**
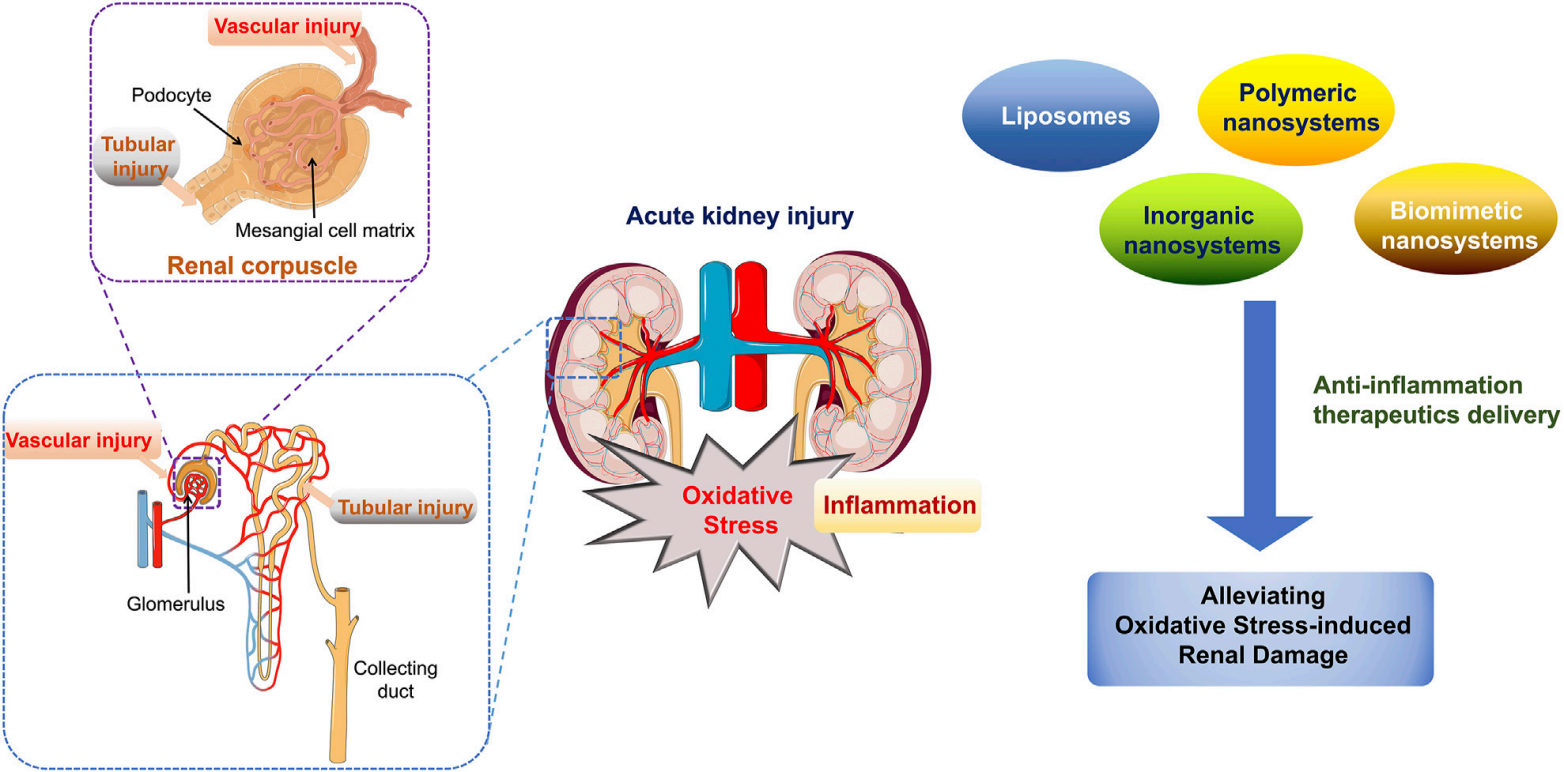
Schematic illustration of biomedical application of various nanosystems in AKI management that alleviate oxidative stress-induced renal cell damage.

Since the kidney is the most important organ for elimination and excretion, many potential therapeutic agents cannot achieve a desired therapeutic effect because of their poor pharmacokinetics and short retention time in the kidneys (Aymanns et al., 2010). Therefore, therapeutic management strategies for AKI focus on the development of targeted therapeutics for the repair and regeneration of injured renal tubule cell (Liu C.-P. et al., 2019). In this review, we summarized the application of nanodelivery systems in AKI treatment by alleviating oxidative stress-induced renal cell damage and regulating the inflammatory kidney microenvironment. Various types of nanosystems have been designed and applied to improve the pharmacokinetics of drug formation, which could further relieve the burden on the kidneys caused by the final cumulative dose of a drug in conventional treatments (Figure 1). Moreover, the passive or active targeting effect of nanosystems can also reduce the total therapeutic dose and off-target adverse effects to other organs. Through this review, the recent progress in nanodrug-based anti-inflammatory strategies, by regulating intracellular oxidative stress, is emphasized and this may inspire the design of advanced nanodrugs and new promising strategies for AKI management.

## 2 The oxidative stress –related molecular biological mechanism of AKI

The pathophysiology of AKI involves in declining glomerular filtration rate (GFR) mediated by systemic or local hemodynamic alterations, reversible tubular injury, and tubular necrosis (Sharfuddin and Molitoris, 2011). During the process of renal tubular damage, injured tubular epithelial cells (TECs) may lead to apoptosis and necrosis (such as acute tubular necrosis) in severe injury and result in reduced excretion of metabolic waste products. Various disease characteristics, including renal ischemia-related cell damage and necrosis, inflammation and oxidative stress, and nephrotoxic injury, are observed during complex physiological and pathological processes (Bonventre and Yang, 2011). Many studies have demonstrated that a disrupted cellular redox system leads to dramatically increased reactive oxygen species (ROS) levels in renal tubular epithelial cells, which results in abnormal permeability of outer mitochondrial membranes and mitochondrial dysfunction when AKI occurs. Exacerbated oxidative stress induced by excess ROS in damaged mitochondria leads to apoptotic or necrotic cell death, and proinflammatory factors, such as tumor necrosis factor-α (TNF-α), monocyte chemotactic protein-1 (MCP-1), interleukin-8 (IL-8), IL-6, IL-1β, and transforming growth factor β (TGF-β) are subsequently released. Inflammation and chemokines participate in the chemotaxis and activation of leucocytes, which results in aggravated cellular inflammation (Sato and Yanagita, 2018). Simultaneously, inflammatory damage can also lead to structural damage and microvascular endothelial dysfunction in the tubulointerstitium, which leads to increased permeability (Schnaper, 2017). Moreover, injured microvascular endothelial cells and further leukocyte activation interact with adhesion molecules to actuate the immuno-inflammatory cascade. Most importantly, inflammatory effector cells are further activated and trigger the release of large amounts of inflammatory mediators, which leads to an amplified inflammatory response and further injury to renal tubular epithelial cells; this becomes a vicious cycle that ultimately leads to kidney dysfunction (Jang and Rabb, 2015; Chew and Hwang, 2019; Han and Lee, 2019).

In order to explore effective clinical therapeutic schedules, numerous studies have been reported to reveal the inflammatory response and cell signal transduction pathway during AKI, such as mitogen-activated protein kinase (MAPK), Toll-like receptor (TLR), and NF-κB signal pathway, which were considered as important inflammatory signaling after renal injury (Table 1).

**TABLE 1:** Signal pathways for oxidative stress regulation in acute renal injury (AKI).

Signal path	Kidney structure/cell	Regulatory role in AKI process	References
TFAM	Mitochondria of renal tubular epithelial cells	Mitochondrial function needs mtDNA very much. The maintenance of mtDNA mainly depends on mitochondrial transcription factor (TFAM). Under pathological conditions, blocking of TFAM will lead to mtDNA depletion and mitochondrial damage	Zhao et al. (2021a)
MAPK/JNK	Proximal renal tubular epithelial cells	Overexpression of P75NPR will increase ROS production and phosphorylation of P38MAPK/JNK pathway, resulting in oxidative stress and cell damage	Wang et al., (2020a)
TLR4/NLRP3	Renal tubular epithelial cells	LPS can activate Toll-like receptors, produce NLRP3 inflammatory corpuscles, induce downstream signal cascades and the expression of inflammatory cytokines, and lead to oxidative stress, activate renal tubular epithelial cells and cause functional damage, and then lead to renal microcirculation disorder and insufficient perfusion, and finally form AKI	Yifan et al. (2020)
NF-κB/Nrf2	Renal tubule	The expression of most antioxidant enzymes is regulated by antioxidant response elements and activated by nuclear factor E2 related factor 2 (Nrf2). In addition, Nrf2 can regulate NF- κ B. Inhibit the expression of inflammatory mediators in renal tissue, such as cytokines, chemokines, adhesion molecules, matrix metalloproteinase-9 (MMP-9), cyclooxygenase-2 (COX-2) and inducible nitric oxide synthase (iNOS), so as to play a protective role in kidney	Zhang et al. (2023a)
PI3K-AKT	Nucleus	PI3K/AKT is an important pathway involved in oxidative damage and inflammation. PI3K/AKT is one of the upstream pathways to regulate Nrf2 and NF- κB. Phosphorylated PI3K and AKT proteins control the activity of downstream substrates *via*. Many compounds can activate Nrf2 or NF -κB through PI3K/AKT pathway, involved in oxidative stress and inflammatory process, to protect cells or tissues from damage	Hu et al. (2017a)
ROS-JNK	Renal tubular epithelial cells	ROS/JNK signaling pathway plays a crucial role in stress response. ROS can activate JNK through apoptotic signal-regulated kinase 1 (ASK1), Src kinase, glutathione s-transferase π (GST π), mixed lineage kinase 3 (MLK3), receptor interaction protein (RIP), tumor necrosis factor receptor-related factor 2 (RAF2) complex and mitogen-activated protein kinase phosphatases (MKPs), mediate JNK pathway, and induce renal injury	Wang et al. (2016)
Nrf2/HO-1	Renal tubular epithelial cells	It is an important pathway involved in oxidative stress in the body. Nrf2/HO-1 pathway is activated at the early stage of renal ischemia/reperfusion injury. Inhibition of oxidative stress in initial cells, was demonstrated with effect of anti-apoptosis, anti-inflammatory, anti-oxidation, thereby alleviating AKI damage	Pei et al. (2020)
P-selectin/PSGL-1	Glomerular vascular endothelial cells	P-selectin/PSGL-1 activates the inflammatory effect by mediating the activation of MAPK signal pathway, leading to inflammatory injury and dysfunction of endothelial cells, and then causing renal injury	Qiu et al. (2014)

## 3 The treatment of oxidative stress regulation for AKI

The restoration of renal perfusion and alleviation of intrarenal causes of AKI are the clinical guidelines for managing patients with AKI. Therapeutic options are required based on the different causes of AKI (Alge and Arthur, 2015; Gonsalez et al., 2019). However, there are currently no specific therapeutics for the prevention or treatment of AKI. Numerous studies have revealed that the pathogenesis of AKI involves a variety of pathological mechanisms, and hemodynamics and oxygen metabolism, inflammation, cell metabolism and oxidative stress, apoptosis, and pathways involved in cytothesis and fibrosis are potential therapeutic targets. This indicates that therapeutic agents for these pathological mechanisms and specifically targeting these signaling pathways may have potential applications for the prevention and management of AKI (Chen and Busse, 2017; Pickkers et al., 2022).

Recent studies have suggested that direct or indirect inflammation can significantly alleviate kidney injury in AKI animal models, which is manifested in a relative decrease in serum creatinine levels and a reduction in renal tubular necrosis (Andrade-Oliveira et al., 2015; Han and Lee, 2019). For instance, human recombinant alkaline phosphatase is an endogenous detoxification enzyme with anti-inflammatory effects. In a phase II trial of patients with sepsis-related AKI, it significantly improved long-term renal function and reduced mortality (Lazzareschi et al., 2022). Moreover, Sirtuin 1 (SIRT1) can inhibit the expression of inflammatory genes and reduce inflammatory tissue injury (Fan et al., 2013). Preclinical studies indicate that NAD^+^-dependent SIRT1 displays protective effects during inflammation-related renal injury by regulating oxidative metabolism in organisms. Therefore, improved SIRT1 activity protects and restores renal function by supplementing the precursor of NAD^+^ (Nicotinamide Riboside and Pterostilbene). It is important to note that this is an ongoing Phase II clinical trial (NCT04342975) (Guan et al., 2017; Chini et al., 2021).

Inflammation is accompanied by oxidative stress, and excess ROS are considered one of the key factors in AKI that provoke oxidative stress and excessive inflammatory response. Recent studies have revealed that the scavenging of ROS in the kidney can effectively regulate the microenvironment of injured renal tubular epithelial cells and alleviate oxidative stress-mediated pathological progression of AKI (Okamura and Pennathur, 2015). For instance, N-acetylcysteine (NAC) supplementation significantly reduced the incidence of AKI and overall mortality after cardiac surgery (Brown et al., 2009; Magner et al., 2022). In preclinical research, various natural antioxidant active ingredients, such as α-lipoic acid, curcumin, and selenium, can scavenge ROS through redox actions to balance intracellular redox. However, these active ingredients have not obtained satisfactory therapeutic effects during *in vivo* application, which is attributed to their rapid metabolism and excretion. As a result, they were seldom precisely delivered to injured kidney tissue and failed to protect endothelial and tubular cells (Iglesias et al., 2013; Jiang et al., 2013; Shanu et al., 2013; Trujillo et al., 2013; Cai et al., 2022).

Increasing evidence shows that the mitochondria produce approximately 90% of ROS in cells (Murphy and Hartley, 2018). Mitochondrial ROS in the local microenvironment of fragile tissues (such as kidneys) may initiate mitochondrial DNA (mtDNA) damage and activate inflammatory cascade reactions and promote end-stage organ damage (Ishimoto and Inagi, 2015; de Seigneux and Martin, 2017; Zhao M. et al., 2021). Among the agents acting on the mitochondria, Szeto-Schiller (SS) peptides are potential mitoprotective compounds that selectively bind to the cardiolipin of the inner membrane of mitochondria and protect against mitochondrial dysfunction. SS-31 is a small peptide that accumulates in the mitochondria as an ROS scavenger and can be rapidly transported to the heart, kidneys, lung, brain and other high perfusion organs *via* intravenous, intraperitoneal or subcutaneous administration (Birk et al., 2013). Preclinical studies have shown that SS-31 exhibits a specific effect on improving the mitochondrial function of renal tubular epithelial cells and is a potential candidate for AKI treatment (Szeto et al., 2011; Hou et al., 2016).

The renal glomerular filtration function based on molecular size and/or charge impedes the delivery of therapeutic agents after renal injury. The clinical treatment of AKI still mainly depends on renal replacement therapy (RRT) although the mortality risk is increased. Therapeutic strategies with enhanced accumulation of therapeutic targeted renal tubular cells are urgently needed in the clinic. With the recent emergence and progress of nanotechnology, nanomedicine with unique physicochemical properties could prolong circulation time, enhance the efficiency of targeted delivery, and elevate the accumulation of therapeutics in crossing the glomerular filtration barrier, which indicates comprehensive application prospects in the prevention and treatment of AKI (Huang Y. et al., 2021; Yu et al., 2021). Nano delivery systems as drug carriers display the diversity and specificity in reduced cytotoxicity, controllable biodistribution and pharmacokinetics, which could be applied in AKI therapy, as well as other renal disease management and diagnosis (Geo et al., 2022). Furthermore, a variety of nanomaterials with unique ROS-scavenging capabilities have also been applied for AKI treatment, and maintain the spatiotemporal equilibrium of biological redox systems, which has evoked nanocatalytic strategies for the regulation of ROS-related inflammation (Huang X. et al., 2021; Zhang C. et al., 2021; Dai et al., 2021).

Nanodrugs display not only targeted delivery and accumulation at specific regions but also regulate the pharmacokinetics and control the drug release in the disease microenvironment (Liu et al., 2023). Commonly, there are three kinds of targeting delivery strategy for kidney injury: 1) Passive targeting: Nanoparticles with a convertible size and surface charge are realized the delivery to renal mesangium (Li and Kataoka, 2021). Nanoparticles of a certain size can balance the affinity of proximal tubules, while biotinylated negative charge groups are easier to intercept in the kidney (Liang et al., 2016; Guo et al., 2017); 2) Active targeting: renal targeting is achieved by the specific combination of receptors on the surface of kidney cells and their ligands. For example, vascular adhesion molecule-1 is a cell surface receptor expressed on podocytes, which can promote receptor-mediated endocytosis for nanocarriers (Visweswaran et al., 2015); 3) Prolonging the circulation time of nanoparticles *in vivo*: for example, the exposure of enzymes/proteins to the compact folded structure of origami DNA is reduced, liver isolation, the opportunity of origami nanostructures with negative electricity to form protein halos is reduced, the uptake in the spleen and liver is reduced, and the excretion and accumulation of kidney are improved. In addition, it is also related to the water solubility and fat solubility, flexibility and rigidity, and density of nanoparticles (Jiang et al., 2018).

## 4 The application of nanosystems for oxidative stress regulation in AKI treatment

**FIGURE 2:**
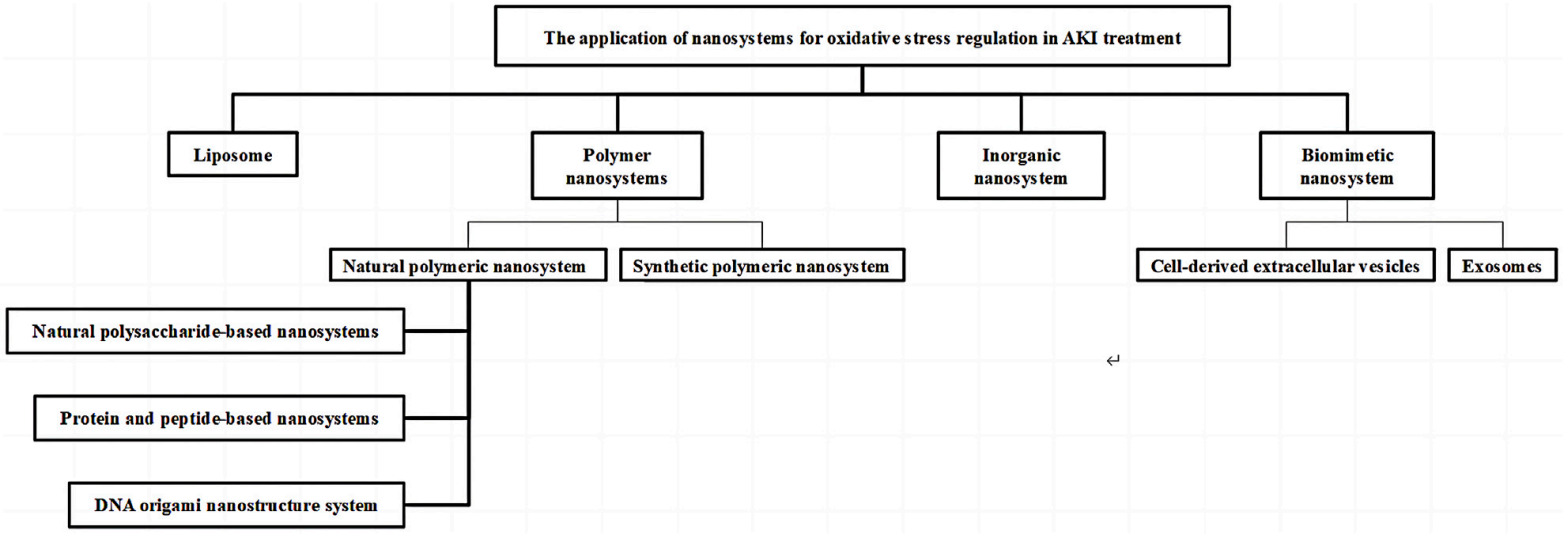
Classification of drug delivery systems for AKI.

Recently, various types of nanosystems with controllable physical and chemical characteristics of size, charge, shape and surface ligand modification have been designed and fabricated to enhance the targeting delivery ability and improve therapeutic efficiency to improve the bioavailability of AKI therapeutic agents (Geo et al., 2022; Chen et al., 2023). In this section, liposomes, polymeric nanosystems, inorganic nanosystems and biomimetic nanosystems based on nanotechnology are explored as treatment strategies for AKI by alleviating the damage of oxidative stress to renal cells (Figure 2).

### 4.1 Liposomes and lipid nanoparticles

Liposomes are carrier systems used to deliver various therapeutic drugs in the research of various nanocarriers (Xing et al., 2016). The hydrophilic and hydrophobic chambers of liposomes enable them to have a high drug-loading capacity. Moreover, special targeting properties could be achieved when modified with ligands such as monoclonal antibodies (Cheng et al., 2022). For example, S.A. Asgeirdottir et al. prepared immune liposomes containing E-selectin antibodies and delivered the multifunctional anti-inflammatory drug dexamethasone (DXA) to inflamed glomerular endothelial cells, which not only selectively inhibit the expression of pro-inflammatory genes to reduce the renal damage caused by glomerulonephritis, but also prevented systemic side effects (Asgeirsdóttir et al., 2007). The high instability of miRNA in systemic circulation and enzyme degradation can also be solved by loading it into liposomes. The overexpression of this liposome in the kidney can significantly reduce the NF-kB-based inflammatory response of kidney cells induced by cisplatin (CDDP), and at the same time, by controlling the process of cell necrosis, prevent the release of endogenous inflammatory and proinflammatory components of cells after cell membrane rupture, thus reducing the severity of renal injury (Zhang S. et al., 2020).

Lipid nanoparticles refer to microcapsules with a much smaller particle size than liposomes, which can improve the solubility and biocompatibility of hydrophobic agents and have the flexibility of lipid modification. Peng Zhang et al. found that nanoliposomes have better renal targeting and can quickly enter the renal cell tissue through the intercellular space, similar to human fatty acids, and then efficiently and quickly enter the organelles inside renal cells. Additionally, they have no biological toxicity, do not produce allergic reactions, and reduce cell apoptosis to prevent renal injury caused by contrast agents (Zhang P. et al., 2021). Ligand-modified solid lipid nanoparticles also exhibited site-specific accumulation and controllable drug release profiles. Sialic acid (SA) is a ligand with a high affinity for the inflammatory vascular endothelium and significantly improves the therapeutic effect of dexamethasone-loaded solid lipid nanoparticles on renal ischemia‒reperfusion injury (IRI)-induced AKI. This nanosystem reduced the levels of superoxide dismutase (SOD) and glutathione (GSH), increased the activity of malondialdehyde enzyme, and decreased the level of oxidative stress of AKI. At the same time, it can also reduce the side effect of osteopenia caused by DXA during treatment (Hu et al., 2017c).

### 4.2 Polymeric nanosystems

Polymeric carriers are made of natural macromolecular materials or synthetic polymer materials through simple processes and are the most widely used drug carriers (Kang et al., 2016). Therapeutic drugs are covalently linked on polymeric materials or embedded in nanoparticles assembled by polymeric materials and alteration of the solubility and metabolism process occurs *in vivo*. Polymer nanocarriers have advantages of high drug encapsulation efficiency, good stability, controllable drug release, and protection of drugs from body fluids and enzymes (Yin et al., 2020).

#### 4.2.1 Natural polymeric nanosystems

##### 4.2.1.1 Natural polysaccharide-based nanosystems

Natural polysaccharide polymers are considered as ideal carriers and scaffolds in the field of drug delivery and tissue engineering because of their good biocompatibility and biodegradability (Yang et al., 2022). Low molecular weight chitosan (LMWC) is a natural linear polysaccharide generated from the deacetylation of chitin. Its main components are D-glucosamine and N-acetyl-D-glucamine. LMWC, as a drug carrier loaded with the antioxidant curcumin (CUR), can target the transport of CUR to renal tubular epithelial cells, which is conducive to drug aggregation in the kidneys. Compared with free drugs, chitosan-loaded drugs can prolong the circulation time of drugs *in vivo*, improve the distribution and accumulation of CUR in tissues, and improve the bioavailability of drugs through aggregation in target epitopes (Wang D.-W. et al., 2021). C. X. Yang designed chitosan nanoparticles to deliver siRNA to treat renal injury caused by urinary obstruction (UUO). Chitosan/siRNA nanoparticles can eliminate cyclooxygenase-2 (COX-2) in macrophages, and thereby reduce renal tubule injury, inflammation, cell apoptosis and oxidative stress to alleviate UUO-induced renal injury. This study targets the phagocytic activity of macrophages and at the same time avoids the serum instability of polymers. Through the homing property of macrophages, siRNA can be specifically gathered into the damaged kidney. Only a low dose of siRNA can achieve a therapeutic effect, and this is also a new treatment method for AKI (Yang et al., 2015). Hyaluronic acid (HA) and chitosan were prepared in nanoparticles by electrostatic composite action, which is used to load mitochondria-targeting antioxidant peptide SS-31 for AKI treatment. As HA is a natural polysaccharide with good biocompatibility and biodegradability, it can accumulate in tissues overexpressing CD44, such as renal tubular epithelial cells repaired after injury and vascular endothelial cells. This nanosystem increases the biological distribution of SS-31 in kidney tissue overexpressing the CD44 receptor and achieves effective delivery. Therefore, in the AKI model, it can reduce the degree of oxidative stress and inflammatory damage in renal tubule tissue and prevent diseases caused by apoptosis and necrosis of renal tubules (Liu D. et al., 2019).

**FIGURE 3:**
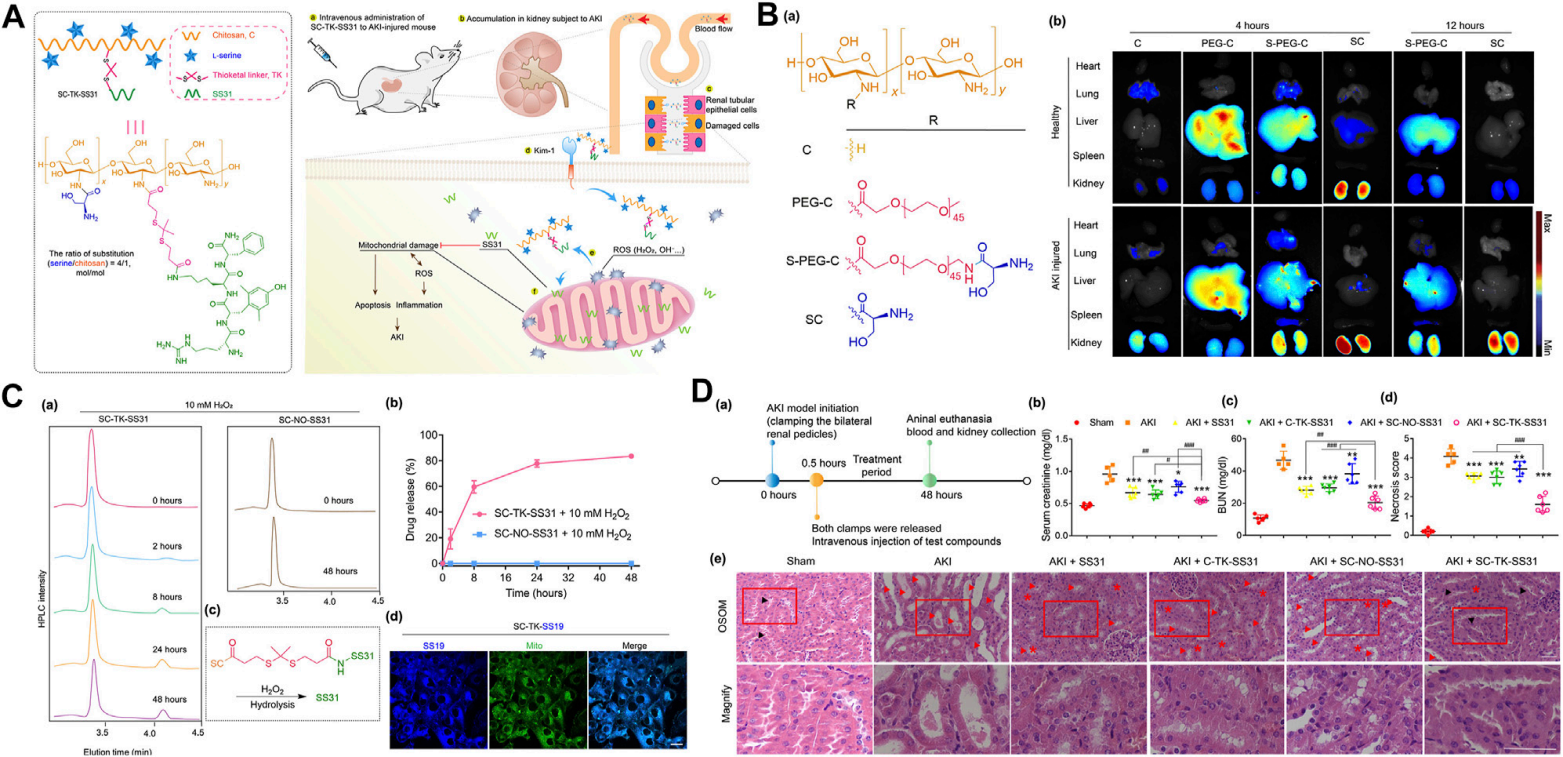
**(A)**Schematic illustration of the design and structural of ROS cleavable polysaccharide drug precursor SC-TK-SS31, and the principle for targeted AKI treatment; **(B) (a)** the design and structural of L-serine-modified chitosan (SC) and its analogues, **(b)** Fluorescent images of heart, lung, liver, spleen and kidney of mice at 4 or 12 h after intravenous injection of various of Cy5-labeled SC and its analogues; **(C) (a)** The absorption peak (220 nm) of SC-TK-SS31 monitored by HPLC in the presence of 10 mM H_2_O_2_, **(b)** the release curve of SC-TK-SS31 and SC-NO-SS31 dissolved in H_2_O_2_ (detected by HPLC), **(c)** Schematic diagram of SS-31 synthesis **(d)** Confocal image of HK-2 cells induced by H_2_O_2_ incubated with SC-TK-SS19 for 12 h. **(D) (a)** the schedule for the blood and kidney collection 48 h after intravenous injection of different drugs for AKI mice, **(b)** Determination of serum creatinine, **(c)** blood urea nitrogen (BUN) and **(d)** necrosis score in AKI mice after different treatments, **(e)** hematoxylin eosin (H&E) staining of renal tissue after different treatment. Adapted with permission from (Sci. Adv. 2020, 6 (41), eabb7422.) (Liu D. et al., 2020).

Additionally, because of the specific interaction between the biomarker molecule of kidney injury (Kim-1) and serine, injured renal tubular epithelial cells targeting L-serine-modified chitosan (SC) was synthesized. The mitochondrial targeted antioxidant SS31was conjugated with SC through ROS cleavable thioketal bonds to prepare a polysaccharide drug precursor (SC-TK-SS31) that can be accurately targeted step by step, combine specific kidney distribution with ROS-responsive drug release behavior, endow the carrier with the ability to target AKI damaged organs (kidneys), cells (renal tubular epithelial cells) and organelles (mitochondria), and multiple combinations enhance the therapeutic effect of SS31 on AKI (Figure 3) (Liu D. et al., 2020).

##### 4.2.1.2 Protein and peptide-based nanosystems

**FIGURE 4:**
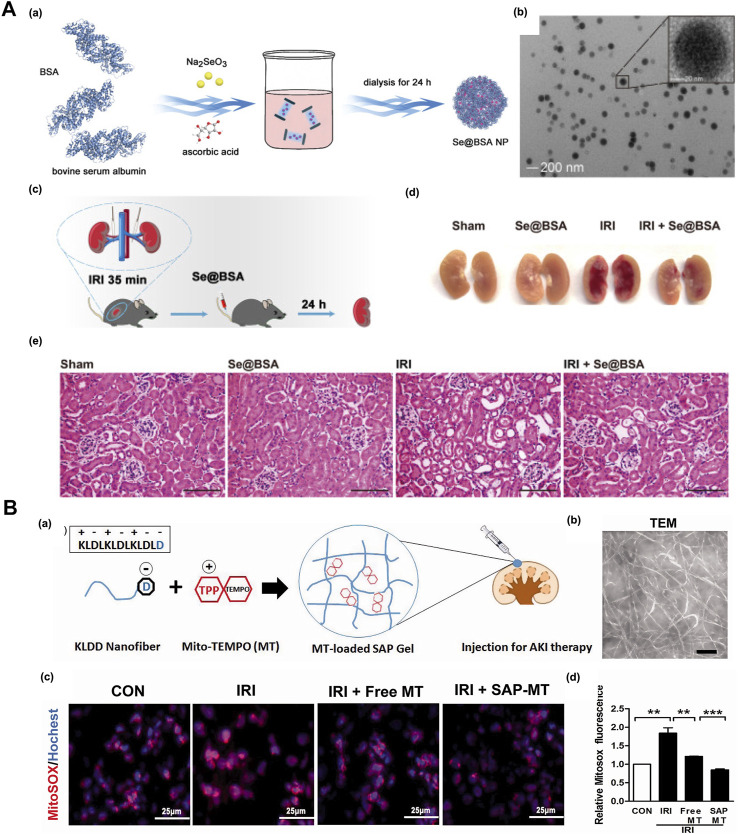
**(A) (a)** The scheme for synthesis of Se@BSA NPs, **(b)**Se@BSA NPs transmission electron microscope image. The region of interest is enlarged in the illustration, where the bare scale indicates 20 nm, **(c)** Schematic diagram of A)IRI-AKI mouse model establishment, **(d)** Selenium content in kidney of IRI-Akiaki mice. The results are shown as mean standard deviation, **(e)** HE staining of the kidneys of AKI mice with Se@BSA NPs treatment. Adapted with permission from (Theranostics. 2022, 12 (8), 3882–3895) (Wang et al., 2022); **(B) (a)** Schematic illustration of Mito TEMPO-loaded KLDD peptide hydrogel for AKI treatment, **(b)** TEM images of KLDD nanofibers, **(c)**The production of renal mtROS was detected by MitoSOX staining after frozen sections, **(d)** Evaluation of renal mtROS level staining. Adapted with permission from (Drug Delivery. 2018, *25* (1), 546–554) (Zhao et al., 2018).

Proteins/peptides have characteristics of low immunogenicity, low toxicity and good biocompatibility and have unique advantages as drug delivery systems. The protein carrier can also reduce the aggregation of nanoparticles. Bovine serum albumin (BSA) is used to improve the stability of selenium nanoparticles (SeNPs); the good biocompatibility will not cause toxic biological reactions and is also a simple and accessible material. Studies have proven that newly synthesized nanomaterial selenoprotein nanoparticles (Se@BSANPs) can reduce the activity of caspase-1 and the proinflammatory factor IL-1 by upregulating the level of GPx-1 and inhibiting the activation of NLRP3 inflammatory bodies in renal tubular epithelial cells after IRI β and IL-18. Se@BSANPs were enriched in the kidney by intravenous injection because this energetic drug regulates the GPx-1/NLRP3/Caspase-1 pathway, improves the renal injury of AKI mice induced by post ischemia perfusion in a dose-dependent manner, and significantly inhibits the subsequent renal fibrosis transformation caused by AKI. It is also a promising drug for treating AKI (Figure 4A) (Wang et al., 2022).

The G3-C12 (ANTPCGPYTHDCPVKR) polypeptide was designed and found to have a renal targeting function after intravenous injection. Captopril (CAP), an angiotensin converting enzyme inhibitor (ACEI), is connected to the G3-C12 peptide through disulfide bonds to obtain a G3-C12-CAP conjugate with kidney-targeting ability. *In vivo* animal experiments show drug enrichment in the kidney and the inhibition of kidney ACE produced by the combination of the drug and peptide carrier (Geng et al., 2012).

Self-assembling peptide (SAP) can form hydrogels under physiological conditions without additional chemical initiators or UV light-induced cross-linking, and thus it can reduce the toxicity and immune reaction of the carrier during treatment. The anionic polypeptide KLDD is prepared by inserting the C-terminus of the self-assembling KLD-12 polypeptide (n-KLDLKLDLKLDL-c) into aspartic acid. Thee negatively charged KLDD nanofiber hydrogel can interact with positive mitochondria-targeting antioxidant Mito-2,6,6-tetramethylpiperidine-N-oxyl (Mito-TEMPO, MT) to regulate its release profiles *in vitro*. The slow release of MT provided continuous protection for IRI-induced mitochondrial dysfunction mice, which alleviated injury of renal tubular and inflammation, indicating the potential of SAP hydrogel as the antioxidant drugs carrier for renal repair after AKI (Figure 4B) (Zhao et al., 2018).

##### 4.2.1.3 DNA origami nanostructure system

**FIGURE 5:**
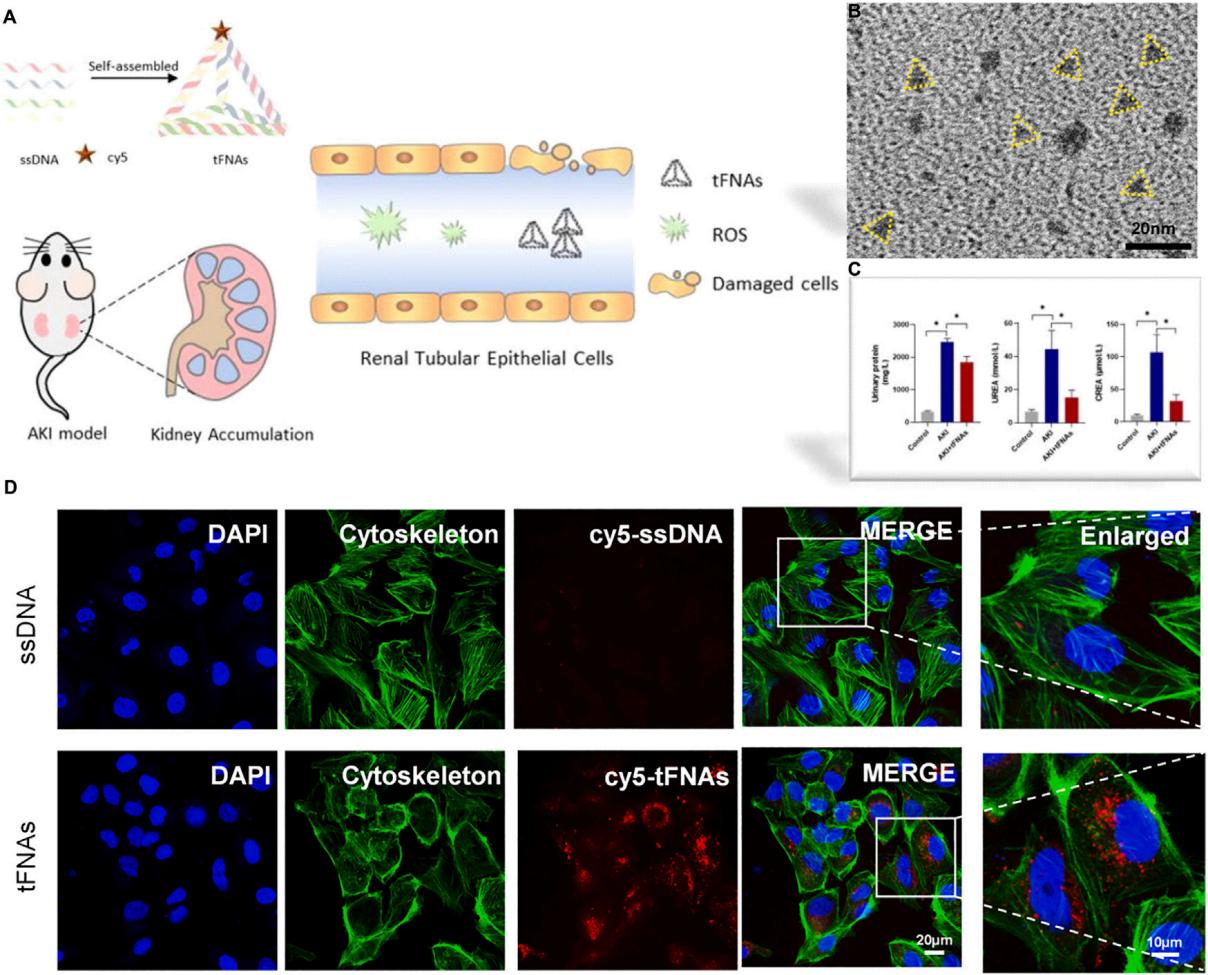
**(A)**Schematic illustration of tFNAs treating RM-induced AKI. TFNAs can reduce the oxidative stress and inhibit apoptosis by targeting renal tubular epithelial cells, showing kidney accumulation and alleviating kidney injury; **(B)** Representative TEM images of tFNAs; **(C)** Analysis of various factors and levels of AKI model mice before and after administration; **(D)** The uptake of Cy5-tFNAs and Cy5-ssDNA by HK-2 cells. Adapted with permission from (Chem. Eng. J., 2021, 413, 127426.) (Zhang Q. et al., 2021).

DNA nanotechnology constructs predictable and programmable nanostructures by using Watson Crick base pairs within and between molecules to design complex structures with different sizes (Linko et al., 2015; Zhang et al., 2015). DNA nanostructures have excellent *in vivo* properties, such as low toxicity, low immunogenicity and good biological stability (Lacroix and Sleiman, 2021; Ma et al., 2021). D. W. Jiang used the single step annealing program to construct three different forms of DNA origami nanostructures (DON): rectangular DON (90 nm × 60 nm), triangular DON (∼120 nm) and tubular DON (∼400 nm). Specifically, rectangular DON has renal protective properties and its efficacy is similar to that of the antioxidant N-acetylcysteine, which can improve AKI and protect renal function from the effects of nephrotoxic drugs (Jiang et al., 2018). Among the DNA nanostructures, the high stability and high accessibility in mammalian cells of tetrahedral skeleton nucleic acid (tFNA) enabled them as the potential therapeutic agent for AKI induced by rhabdomyolysis (RM) (Figure 5A). tFNA is the self-assembly of four single stranded DNA through the principle of base complementary pairing. As shown in the report, tFNA displayed selective targeting and accumulation to the lesion site in RM-AKI mice. The study proved that tFNA can inhibit the apoptosis of renal tubule cell, thereby reducing intracellular oxidative stress and mitochondrial dysfunction. In addition, tFNA displayed the therapeutic effect to alleviate the AKI without any other drug carrying (Zhang Q. et al., 2021).

#### 4.2.2 Synthetic polymeric nanosystems

In addition to natural polymers, synthetic polymers are widely used to prepare nanocarriers. Synthetic polymers can be formed by copolymerization or polycondensation of different monomers or preformed polymers and can precisely control the characteristics of nanoparticles through the modification of various monomer structures so that they can be widely used in drug delivery systems for AKI treatment. Mesoscale nanoparticle (MNPs) prepared from intravenous polyethylene glycol polylactic acid glycolic acid copolymer (PEG-PLGA), which are approved by the FDA, have a particle size of approximately 400 nm. *In vivo* distribution studies show that MNPs can be selectively locate in proximal renal tubular epithelial cells. The study also found that the selective localization of MNPs in renal tubules does not have a negative impact on the kidney (Williams et al., 2015). Furthermore, the nanoparticles are used to deliver a class of NEMO (NF-κB regulator) to the proximal tubule of the kidney. This peptide can block the binding of NEMO and the IKK complex and prevent NF-κB activation and reduce proinflammatory NF by inhibiting NEMO-κB signal generation to prevent ischemic AKI (Han et al., 2020). This MNP is also used to load the B2 adrenergic agonist formoterol and prepare the mesoscale nanoparticles (FNPs) that range between 400 and 500 nm. The nanoparticles can target renal tubular necrosis and fibrosis in mouse models that accumulate in renal tubules and can significantly reduce I/R damage, restore mitochondria and renal function, and avoid cardiovascular toxicity (Vallorz et al., 2022).

Polycation is an effective gene carrier, and the positive charge carried by the polycation can form a complex with gene drugs (pDNA or RNA) through electrostatic interactions and promote the endocytosis of the nanosystem by the effector cells. The linear polymeric chemokine receptor CXCR4 antagonist (PCX) is synthesized by the Michael addition copolymerization of phenyl cyclic derivatives and hexamethylene bisacrylamide. The polymer can effectively deliver p53 siRNA (sip53) to the kidney of AKI models with cisplatin and bilateral ischemia‒reperfusion injury and treat AKI by inhibiting CXCR4 and combining p53 gene silencing to improve kidney function and reduce kidney damag (Tang et al., 2022).

Reactive oxygen-responsive organic polymers (mPEG-TK-PLGA) were prepared by copolymerization of thioacetalinker with PEG and PLGA, which were used to load triphenylphosphine (TPP)-modified cerium nanoparticles and atorvastatin (ATV/PTP-TCeria NPs). This nanoparticle can be passively targeted into the mouse model of acute renal injury caused by sepsis with enhanced vascular permeability, and can passively target renal inflammatory injury to the vascular endothelium, protect the mitochondrial structure, and reduce the apoptosis and necrosis of renal tubular cells by reducing the oxidative stress and inflammatory reaction (Yu et al., 2020).

Perfluorocarbon nanoparticles (PFC NP) are a blood substitute approved by the FDA. The Proline-Phenylalanine-Arginine-Chloromethyl-Ketone (PPACK) polypeptide-conjugated PFC nanosystem formed by chemically coupling the thrombin inhibitor PPACK polypeptide can prevent thrombin signal transmission and microvascular thrombosis, limit inflammatory signal amplification, protect the renal tubule microstructure and reduce damage in atherosclerosis and AKI models (Vargas et al., 2021).

### 4.3 Inorganic nanosystems

Inorganic nanomaterials mainly refer to nanosystems that are constructed of inorganic materials (such as carbon, mesoporous silicon, calcium, gold, and iron), which have advantages of simple preparation, easy surface modification, a high drug-loading rate, small particle size, adjustable size, a large specific surface area, and good biocompatibility (Yang et al., 2019; Wang X. et al., 2021). Nanoenzyme systems based on inorganic nanomaterials reveal the inherent biological effects and new characteristics of nanomaterials and expand their scope of application. Nanozymes are artificial enzymes that are more stable, have a lower cost, have adjustable catalytic activity, are easy to modify and have other unique advantages over natural enzymes or traditional analog enzymes (Jiang et al., 2019; Cai et al., 2021). Additionally, nanomaterials have excellent physical and chemical properties, such as optics, magnetic fields, thermodynamics and large specific surface areas, which can endow nanozymes with multiple functions and regulate the catalytic activity and surface modification of nanoenzyme surfaces (Yang et al., 2018b; Wang et al., 2020b).

**FIGURE 6:**
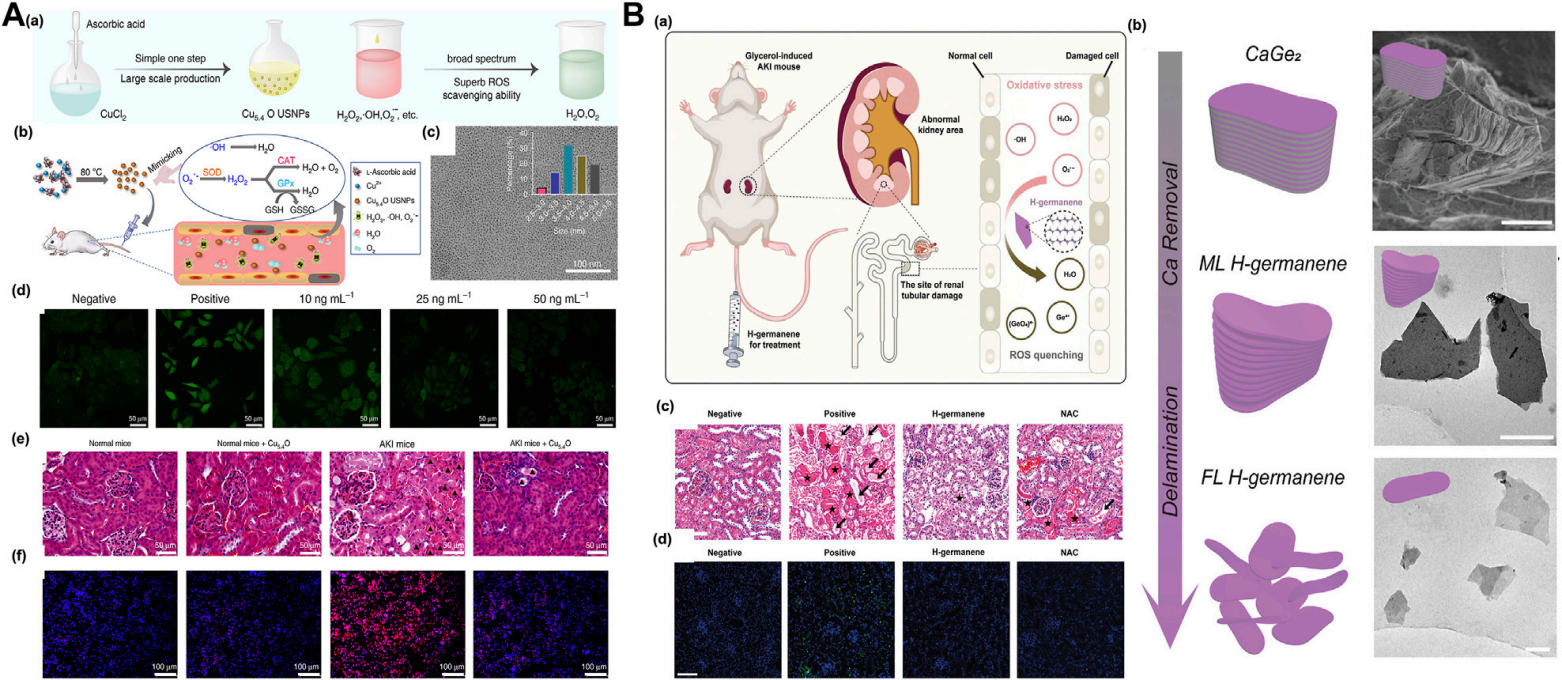
**(A) (a)** Schematic of synthetic process of Cu_5.4_O USNPs (ultrasmall nanoparticles) with broad ROS scavenging property, **(b)** Cu_5.4_O USNPs exhibited SOD, CAT and GPx-mimicking activity present the potentiality for treatment of oxidative stress related injury, **(c)** TEM image of Cu_5.4_O USNPs, **(d)** Representative ROS staining of HEK293 cells with different Cu_5.4_O USNPs treatment, the therapeutic efficacy of Cu_5.4_O USNPs *in vivo*: **(e)** H&E staining of kidney tissues **(f)** Dihydroethidium staining of kidney tissues for each group. Adapted with permission from (Nat. Commun. 2020, 11, 2788.) (Liu S. et al., 2020b); **(B) (a)** The schematic illustration of H-germanene as an ROS-quenching agent accumulated at the damaged renal tubular for AKI treatment, **(b)** characterization of precursor CaGe_2_ and H-germanene nanosheets: synthetic process of the H-germanene (left) and SEM image of the precursor CaGe_2_, TEM images of ML H-germanene and FL H-germanene (right), **(c)** H&E staining of the kidney tissues, **(d)** TUNEL staining of the kidney tissues. Adapted with permission from (Adv. Sci. 2022, 9, 2202933.) (Chen et al., 2022).

Research shows that the Prussian blue (PB) nanoenzyme system based on Prussian blue can effectively eliminate ROS *in vivo* and *in vitro*, and the ultrasmall PB nanoenzyme prepared with natural chitosan as a template can accumulate in the kidney tissue. Because of their excellent enrichment in the kidney and unique multienzyme simulation ability, ultrasmall PB nanoenzyme have shown better therapeutic effects than amifostine in rhabdomyolysis- or cisplatin-induced AKI mouse models (Zhang et al., 2021c). As a broad-spectrum ROS scavenger, self-assembled ultrasmall nanoparticles (FGP nanoparticles) composed of iron ions, gallic acid and polyvinylpyrrolidone can alleviate mouse AKI induced by glycerol and cisplatin (Zhang et al., 2021b). After the combination of Mn^2+^-chelated melanin (MMP) nanoparticles and polyethylene glycol, the obtained coordination assembled MMPP nanoparticles have the ability to eliminate a variety of reactive oxygen free radicals and inhibit the oxidative stress induced by reactive oxygen species (Sun et al., 2019). Ultrasmall Cu_5.4_O nanozymes simulate various biological enzymes and have a broad spectrum and efficient active oxygen scavenging capacity. They can reduce oxidative stress and inhibit NF by eliminating excessive ROS-κB signaling and downregulating proinflammatory factors such as TNF-α and IL-1β. The secretion of IL-6 reduces damage to kidney tissue (Figure 6A) (Liu S. et al., 2020b). Some studies have used the unique redox cycle characteristics of nanoceria, that is, it can cycle between Ce^3+^ (reduction) and Ce^4+^ (oxidation) states, which can improve its ability to scavenge active oxygen. As an antioxidant, citric acid-modified ceria nanoenzymes can effectively protect renal tubular epithelial cells against H_2_O_2_ stimulation *in vitro*. In addition, because of their very small size, cerium dioxide nanoparticles can effectively accumulate in the kidney, clear ROS in the kidneys, and protect kidney cells. Ultrasmall cerium dioxide nanoenzymes can be used as antioxidants to alleviate AKI caused by rhabdomyolysis and show great clinical potential (Zhang D.-Y. et al., 2020). Activation of the Nrf2/Keap1 signaling pathway regulates ROS-related genes by regulating the catalytic activity of cerium oxide nanoenzymes (CNPs) so that they can effectively eliminate ROS in the kidneys (a neutral microenvironment). In the acidic tumor microenvironment, excessive H^+^ interferes with the re-exposure of active catalytic sites, and inert CNPs no longer interfere with the generation of ROS mediated by chemotherapy for cancer treatment. Catalytic activity of the microenvironment response and adjustable CNP regulation of ROS can open up a new way for patients (especially patients receiving chemotherapy) to prevent AKI (Weng et al., 2021).

The 2D-H germanium nanosheet with flaky DNA geometry has a broad-spectrum free radical scavenging effect, which can effectively remove hydrogen peroxide (H_2_O_2_), superoxide anions (O_2_•^-^) and hydroxyl radicals (•OH). Because of their geometric structure that is similar to DNA, such new 2D nanomaterials can effectively accumulate in the kidneys, improve the side effects of conventional antioxidant drugs, such as N-acetylcysteine and amifostine, and avoid rapid metabolism, low utilization and low efficacy while having no significant toxicity for treating AKI (Figure 6B) (Chen et al., 2022). A new nanoenzyme antioxidant system based on ultrathin Ti_3_C2-PVP nanosheets (TPNSs) also has biodegradability triggered by enzymes/ROS. The nanoenzyme system shows a broad-spectrum ROS-scavenging ability, can inhibit the NF-κB signaling pathway, is used to inhibit the inflammatory reaction induced by oxidative stress and can treat related diseases caused by AKI and other ROS (Zhao X. et al., 2021).

### 4.4 Cell-derived extracellular vesicles and exosomes

Cell-derived extracellular vesicles and exosomes are extracellular membranous lipid vesicles that are secreted by cells. They play critical roles in cell signaling transportation by transferring proteins, RNA, DNA, and other substances for intercellular communication and become increasingly popular topics in disease diagnosis, treatment and prevention (Richter et al., 2021). Currently, exosomes are considered disease biomarkers and prognostic factors and exhibit further potential as delivery vehicles for gene and chemotherapy drugs with important clinical significance (Li et al., 2020).

**FIGURE 7:**
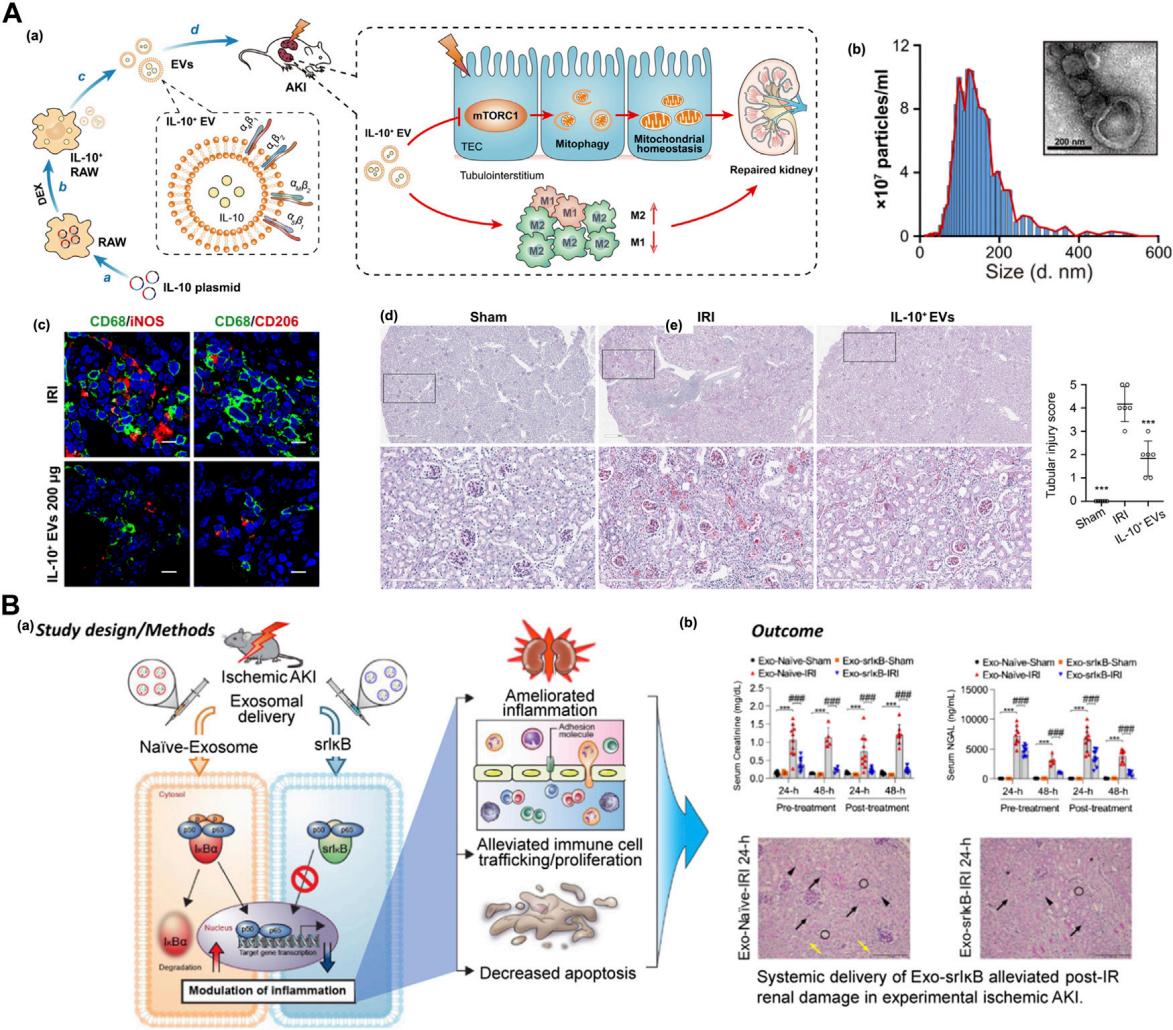
**(A) (a)** Schematic of the IL-10-loaded extracellular vesicles (IL-10 + EVs) with property of reducing inflammatory cell infiltration by mTOR inhition for targeted therapy of IRI-AKI, **(b)** the nano-/micro-morphology of IL-10^+^ EV and the size distribution, Scale bars, 200 nm, **(c)** the immunofluorescence images of M1 (CD68^+^iNOS^+^) phenotypes and M2 (CD68^+^CD206^+^) phenotype macrophages in kidney section with different treatment, Scale bars, 10 μm, **(d)** periodic acid–Schiff (PAS) staining of renal tissue after different treatment to evaluate tubular injury score. Adapted with permission from (Sci. Adv. 2020, 6, eaaz0748) (Tang et al., 2020); **(B) (a)** The schematic illustration of exosome-based anti-inflammatory super-repressor IκBα protein (SrIκB) reduce the off-target effects by targeting the cytoplasmic inflammatory signaling directly, which is differ from the conventional inhibition of NF-κB upstream signaling; **(b)** the efficacy of the anti-inflammatory exosomes for the treatment of IRI-AKI *in vivo*. In a preclinical IRI-AKI murine model, systemic administration of ILB-202 significantly reduced the AKI-associated serum biomarkers, BUN (blood urea nitrogen), creatine, and NGAL (neutrophil gelatinase-associated lipocalin). Adapted with permission from (Kidney Int. 2021, 100 (3), 570–584.) (Kim et al., 2021).

A recent study found that IL-10-loaded extracellular vesicles (IL-10^+^ EVs) were produced from RAW macrophages by plasmid transfection and dexamethasone intervention. This IL-10^+^ EV, as part of the IL-10 delivery platform, could accumulate in tubular epithelial cells and macrophages after IRI. The therapeutic efficacy of IL-10 could be beneficial for maintaining mitochondrial homeostasis by suppressing mTOR signaling, significantly reducing inflammatory cell infiltration and alleviating pathological damage in the kidney, which displayed the potential for targeted therapy of IRI-AKI (Figure 7A) (Tang et al., 2020).

The kidney injury molecule-1 (Kim-1) is a transmembrane glycoprotein on renal proximal tubule epithelial cells whose expression is low in normal kidney tissue. However, the expression of Kim-1 is significantly upregulated in proximal tubular epithelial cells after IRI, which could serve as a novel biomarker for AKI (Rees and Kain, 2008; Vaidya et al., 2010; Boyacıoğlu et al., 2022). LTH peptide, a Kim-1–specific binding peptide, was designed and applied for the engineered modification of red blood cell-derived extracellular vesicles. This biomimetic nanosystem has been utilized to deliver siRNAs of P65 and Snai1 transcription factors to injured tubular epithelial cells. The inhibition of P65 and Snai1 simultaneously attenuated renal inflammation and fibrosis in a murine model of IRI and UUO, and slowed the progression of ischemic AKI (Tang et al., 2021).

ILB-202 (Exo-SrIκB) is an exosome-based therapeutic agent containing the anti-inflammatory superrepressor IκBα protein (SrIκB), which was developed for acute and chronic inflammatory diseases. SrIκB, the main active form of IκBα, was proven to attenuate inflammatory responses in various diseases by inhibiting NF-κB nuclear translocation. ILB-202 reduces off-target effects by directly targeting cytoplasmic inflammatory signaling, which differs from the conventional inhibition of NF-κB upstream signaling (Choi et al., 2020). The ILIAS company demonstrated the efficacy of anti-inflammatory exosomes for the treatment of IRI-AKI *in vivo*. In a preclinical IRI-AKI murine model, systemic administration of ILB-202 significantly reduced the AKI-associated serum biomarkers BUN (blood urea nitrogen), creatine, and NGAL (neutrophil gelatinase-associated lipocalin) (Figure 7B) (Kim et al., 2021).

### 4.5 Modification of nanosystems for active targeting in TKI therapy

In addition to their own specific enrichment effect, nanosystems can also achieve the active targeting drug delivery by surface modification, and thereby enhance the therapeutic effect of various antioxidant damage drugs on AKI (Yang et al., 2018a; Liu et al., 2021).

**FIGURE 8:**
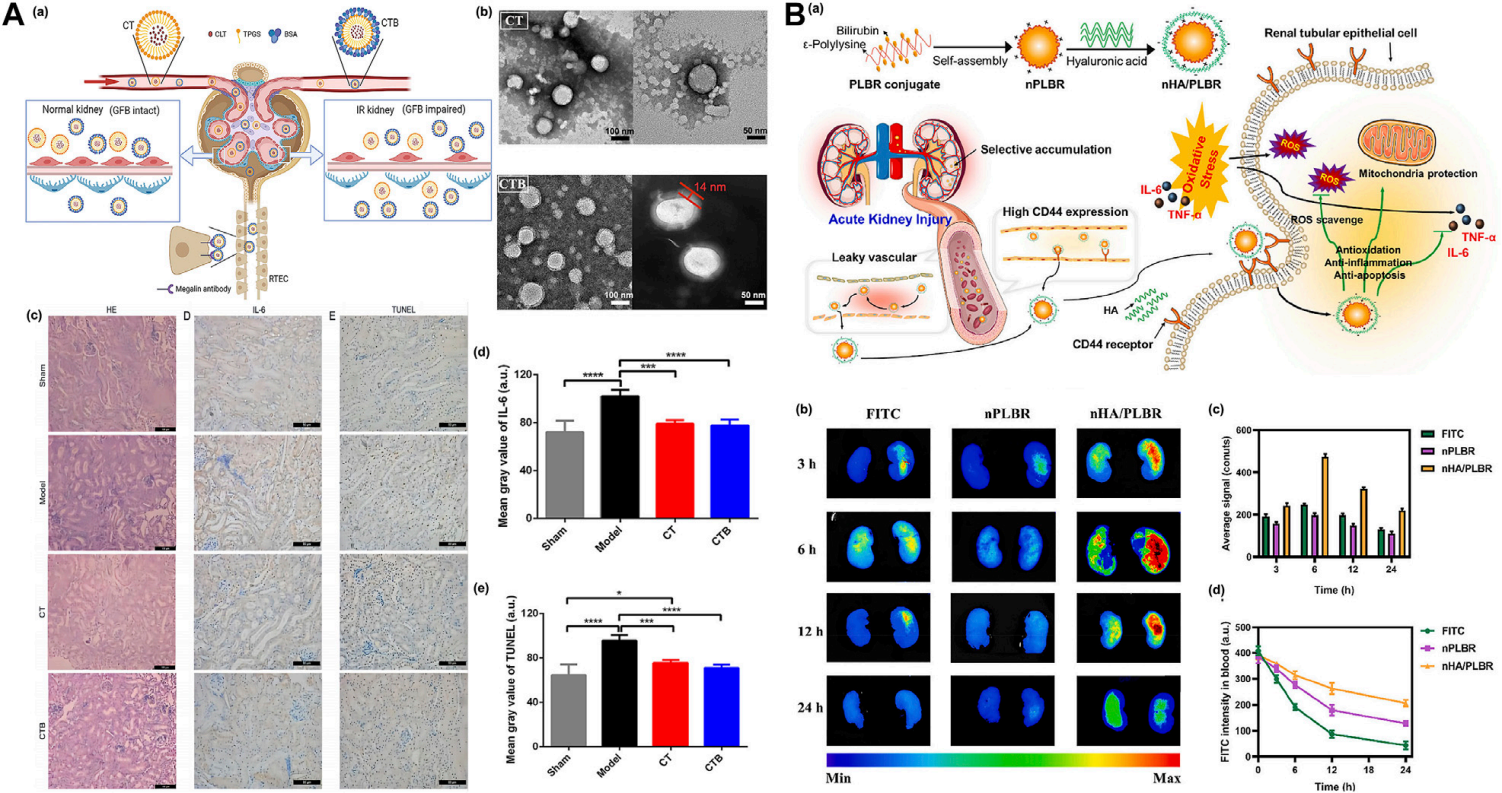
**(A) (a)** Schematic diagram of the action of CLT-TPGS nanocomposite (CT) in IR-AKI mice. GFB, glomerular filtration barrier; RTEC, renal tubular epithelial cells, **(b)** Transmission electron microscope (TEM) images of CT or CTB, **(c)** The results of H-E staining, representative images of IL-6 expression, and TUNEL method were used to detect the apoptosis of renal cells in different groups, **(d)** Semi-quantitative results of IL-6 expression, **(e)** Semi-quantitative results of renal cell apoptosis. Adapted with permission from (J. Control. Release 2022, 349, 401–412.) (Qin et al., 2022); **(B) (a)** nHA/PLBR can accumulate in damaged renal tubular epithelial cells by targeting CD44, **(b)** Renal fluorescence intensity of AKI rat models treated with three different drugs, **(c)** Fluorescence intensity analysis, **(d)** Comparison of FITC fluorescence intensity in blood of mice in each group after administration. Adapted with permission from (J. Control. Release 2021, 334, 275–289.) (Huang Z.-W. et al., 2021).

Renal tubular epithelial cells (RTECs), as the main undertaker of the renal reabsorption function, are one of the main sites of AKI. These are based on ε Physicochemical properties of polylysine and design and synthesis of natural peptide bond linked kidney-targeting peptide (Lysine-Lysine-Glutamic acid-Glutamic acid-Glutamic acid)3-Lysine, named (KKEEE)_3_K), which shows ideal pharmacokinetic characteristics and a high affinity for the kidneys (Wischnjow et al., 2016). This optimized polypeptide can bind to the receptor giant protein on the surface renal tubule cells to achieve targeted delivery. The amphiphilic micelles self-assembled by (KKEEE)_3_K peptide conjugated DSPE-PEG(2000)-amine with excellent renal proximal tubule cells targeting property, can principally reduce the off-targeting aggregation. (Wang et al., 2018). Although ligand-modified nanoparticles are used in the RTEC targeting strategy after systemic administration, NPs easily bind non-specific plasma proteins, which greatly weaken the targeting efficiency and stability of NPs. ShuoQin et al. buried tripterine (CLT) in D-α-tocopherol polyethylene glycol 1,000 succinate (TPGS) to form a nanocomposite core (CT) loaded with CLT, and then BSA was attached to the CT surface to afford a complete albumin corona without obvious denaturation (CTB). The experiment proved that the particle size distribution of CTB was uniform and stable both *in vivo* and *in vitro*, and CTB could be actively internalized in RTEC cells through macroprotein receptor-mediated endocytosis. CTB showed enhanced tubular-specific distribution and targeting in mice. In addition, pharmacodynamic studies *in vivo* further support the conclusion that CTB can reduce the IRI-AKI effect without obvious systemic side effects (Figure 8A) (Qin et al., 2022).

Vascular endothelial cells (VEC) are lined in the entire circulatory system from the heart to the capillaries and have unique vascular biological functions. In acute renal injury, glomerular vascular endothelial cells are damaged. Therefore, targeting anti-inflammatory drugs to endothelial cells with damaged glomerular blood vessels also reduces apoptosis and necrosis induced by oxidative stress/inflammation and can be used for the treatment of AKI. Selectin is a glycoprotein located in the membrane of endothelial cells (ECs) and is mainly used to participate in the recognition and adhesion of leukocytes and endothelial cells. After endothelial cells are activated by proinflammatory factors, the expression levels of P-selectin and E-selectin are highly upregulated. Blocking these two targets may have significant renoprotective effects. Therefore, selectin is considered an ideal target for drug delivery and the effective treatment of inflammatory, injury-related diseases such as AKI. Fucoidan is composed of L-fucose and sulfate, which can specifically recognize and adhere to inflammatory injured vascular endothelial cells that highly express P-selectin. Using fucoidan as the target, the kidneys target octyl succinic anhydride fucoidan/curcumin micelles (OSA Fucoidan/CUR) (Shu et al., 2021). Q. Kang prepared sialic acid polyethylene glycol dexamethasone (SA-PEG-DXM) coupling micelles. Because SA can specifically bind E-selectin, SA-modified drugs are more easily absorbed by damaged vascular ECs so more drug-loaded micelles accumulate in the kidneys of AKI model mice, which is also a new treatment strategy for AKI (Hu et al., 2017b). Hyaluronic acid (HA)-wrapped ε-polylysine bilirubin conjugate nanoparticles (nHA/PLBR) was designed and prepared by self-assembling with PLBR and followed by coating with HA though electrostatic interactions. The nanoparticles can accumulate in the injured renal tubular epithelial cells and vascular endothelial cells through the CD44 targeting effect mediated by HA. Bilirubin can regulate the polarization of macrophages to M2 (anti-inflammatory type), reduce oxidative stress/inflammatory induced damage, effectively protect the kidney from serious damage and maintain renal function (Figure 8B) (Huang Z.-W. et al., 2021).

In addition, some nanomaterials with properties of regulating oxidative stress are utilized for AKI management, for instance, ammonium functionalized carbon nanotubes (fCNT) for siRNA delivering siRNA, lecithin emulsion with F_6_H_8_ (perfluorohexane), and TNF-α neutralizing antibody and hepatocyte growth factor assembled hydrogel are reported to repair of damaged kidney tissue, which can inhibit the production of proinflammatory cytokines and the infiltration of macrophages in kidney tissue (Alidori et al., 2016; Tsagogiorgas et al., 2019; Liu S. et al., 2020b).

## 5 Conclusion and perspectives

In conclusion, individualized strategies for the prevention, diagnosis and management of AKI based on different causes and conditions are still a major challenge for clinical research. The incidence of AKI involves hemodynamic alterations, inflammation, and damage to the vascular endothelium and tubular epithelial cells. The dedifferentiation and proliferation of surviving epithelial cells gradually restores the functional integrity of the kidney tubular epithelium, which indicates the inherent self-repair capability of the epithelium, while maladaptive and abnormal repair leads to chronic kidney disease. The design of targeted treatment strategies to alleviate damage and accelerate repair based on a comprehensive understanding of the underlying pathophysiological process of acute kidney injury and repair is urgent.

Therefore, the alleviation of oxidative stress, inflammation and apoptosis in injured kidneys is crucial for AKI management. However, the application of antioxidant ingredients still faces challenges for AKI therapy. Conventional antioxidation treatment seldom delivers antioxidants to renal tubular epithelial cells accurately and fails to combat the cascade reaction of oxidative stress within damaged mitochondria since the kidney is an important excretory organ of the human body. Whether nanodrugs for antioxidant ingredient delivery or nanobiomaterials with free radical scavenging capability are used, nanosystems will still need to optimize their renal targeting efficacy and continuously adjust their metabolism processes *in vivo* to effectively improve drug efficacy and the therapeutic index in the management of AKI. In addition, promoting the stability of highly effective antioxidant-loaded nanosystems and their biosafety within physiological conditions *in vivo* has become an important cornerstone for future nanodrugs with clinical transformation in the personalized treatment of AKI. With a comprehensive understanding of the pathogenesis and molecular biology of AKI, innovative research on molecular pharmacology and nanodrug systems based on nanotechnology will benefit the personalized treatment of AKI with individual causes and conditions. In the future, an intensive investigation of the therapeutic action, pharmacokinetics, toxicology and biological mechanisms of antioxidant nanodrugs would promote relevant clinical trials and the application of clinical AKI management.
